# Antibacterial Mode of Action of β-Amyrin Promotes Apoptosis-Like Death in *Escherichia coli* by Producing Reactive Oxygen Species

**DOI:** 10.4014/jmb.2209.09040

**Published:** 2022-11-09

**Authors:** Giyeol Han, Dong Gun Lee

**Affiliations:** School of Life Sciences, BK 21 FOUR KNU Creative BioResearch Group, Kyungpook National University, Daegu 41566, Republic of Korea

**Keywords:** β-amyrin, apoptosis-like death, reactive oxygen species

## Abstract

β-Amyrin is a pentacyclic triterpene widely distributed in leaves and stems worldwide. The ability of β-amyrin to induce the production of reactive oxygen species (ROS) in microorganisms suggests its potential as an antimicrobial agent. Thus, this study aimed to elucidate the antibacterial mode of action of β-amyrin. We treated *Escherichia coli* cells with β-amyrin and found that it triggered ROS accumulation. Excessive stress caused by ROS, particularly hydroxyl radicals, induces glutathione (GSH) dysfunction. GSH protects cells from oxidative and osmotic stresses; thus, its dysfunction leads to membrane depolarization. The resultant change in membrane potential leads to the release of apoptotic proteins, such as caspases. The activated caspases-like protein promotes the cleavage of DNA into single strands, which is a hallmark of apoptosis-like death in bacteria. Apoptotic cells usually undergo events such as DNA fragmentation and phosphatidylserine exposure, differentiating them from necrotic cells, and the cells treated with β-amyrin in this study were positive for annexin V and negative for propidium iodide, indicating apoptosis-like death. In conclusion, our findings suggest that the antibacterial mode of action of β-amyrin involves the induction of ROS, which resulted in apoptosis-like death in *E. coli*.

## Introduction

Triterpenes are the most abundant natural products investigated to date. Especially, pentacyclic triterpenes are secondary plant metabolites can be easily found in fruit peel, leaves, and stem bark [1]. Their pharmacological effects have been applied to prevent and treat retinal disease, and they have shown high efficiency in wound healing [2, 3]. The antimicrobial effect of pentacyclic triterpenes is originated from their ability to trigger the production of reactive oxygen species (ROS) by microorganisms [4, 5]. Pentacyclic triterpenoids includes α-amyrin and β-amyrin [6, 7] which has been reported to produce mitochondrial ROS that destroy organelles and prevent cell proliferation [5, 8].

The lethal effects of ROS on bacteria have been elucidated [8, 9]. Oxidative stress damages both on the backbone and bases of nucleic acids and plays an important role in the antibiotic-mediated killing of bacteria. When superoxide (O_2_^−^) dismutase, a component of the bacterial defense system, degrades ROS into superoxide (O_2_^−^) and hydrogen peroxide (H_2_O_2_), even a small amount of ROS can induce cellular dysfunctions, including disruption of lipid and protein synthesis and activation of cell death signaling pathways.

Cell death pathways are investigated including necrosis and apoptosis. The cells undergoing apoptosis is not harmful itself and causes no inflammation [10]. Especially, apoptosis is a type of programmed cell death that usually occurs in eukaryotes. However, apoptosis-like death has also been reported in bacteria in recent years [11]. Although the apoptotic activity of β-amyrin has been examined previously , with studies revealing that it exhibits pharmacological effects via diverse processes, the mechanism underlying its antibacterial effect remains unelucidated [5, 12]. Therefore, this study aimed to determine the antibacterial mode of action of β-amyrin-induced apoptosis in *Escherichia coli*.

## Materials and Methods

### β-Amyrin Solution and Cell Culture Conditions

β-Amyrin was obtained from Sigma–Aldrich (USA) and was dissolved in distilled water to a final concentration of 5 μM. *E. coli* (BW 25113) was obtained from the Coli Genetic Stock Center (USA) and its purity was ≥ 95% (High-performance liquid chromatography [HPLC]). The cells for experiments were cultured overnight in Luria–Bertani medium at 37°C. Then, cells were purified with centrifugation in 12,000 ×*g*.

### Determination of Superoxide (O_2_^−^) and Hydroxyl Radical (OH^−^) Generation

ROS generation was determined using dihydroethidium ([DHE], Molecular Probes, USA) for O_2_^−^ detection and 3′-(p-hydroxyphenyl) fluorescein ([HPF]; Molecular Probes) for OH^−^ detection. Briefly, the *E. coli* cells (1 × 10^6^ cells/ml) were treated with β-amyrin (5 μM) or norfloxacin (5 μM). Thereafter, they were incubated for 2 h at 37°C and centrifuged at 12,000 ×*g* for 5 min, following which the supernatant was removed. Finally, phosphate-buffered saline (PBS) was added to the bacterial pellet, and it was dyed with DHE or HPF. The results were assessed using the Shimadzu RF-5301PC spectrofluorophotometer (Shimadzu, Japan) at 490 nm (excitation) and 515 nm (emission) for the DHE-dyed samples and at 510 nm (excitation) and 595 nm (emission) for the HPF-dyed samples.

### Assessment of ROS-Induced Damage to the Bacterial Defense System

ROS destroy the defense system of *E. coli* by causing oxidative damage, and glutathione (GSH) is a hallmark of oxidative stress [13, 14]. A decreased ratio of GSH to glutathione disulfide (GSSG) indicates oxidation degrees, and this can be measured using GSH assay [15]. Briefly, *E. coli* cells (1 × 10^6^ cells/ml) were treated with β-amyrin (5 μM) or norfloxacin (5 μM), and some cell samples were pretreated with thiourea (150 mM) before adding β-amyrin. Subsequently, they were incubated for 2 h at 37°C. Next, at 12,000 ×*g* centrifuge, the cells were separated into supernatant and pellets. Then, 400 μl homogenization buffer (50 mM Tris [pH 7.5], 1 mM phenylmethylsulphonyl fluoride, and 2 mM EDTA) was added and the beads for the cell lysis was added. The samples were shaken for 1.5 min and incubated for 1 min, and this process was repeated four times. The cells were then resuspended in a 5% 5-sulfosalicylic acid solution for protein precipitation and pretreated with 1 μl of 2-vinylpyridine for GSSG measurement, following which the absorbance of samples at 415 nm was measured using a microtiter plate ELISA reader (BioTek Instruments, USA).

### Membrane Depolarization

Changes in membrane potential lead to the release of apoptotic factors, and this can be measured using bis-(1,3-dibutylbarbituric acid)trimethine oxonol (DiBAC_4_(3); Molecular Probes). Briefly, *E. coli* cells (1 × 10^6^ cells/ml) were treated with β-amyrin (5 μM) or norfloxacin (5 μM), and some cell samples were pretreated with thiourea (150 mM) before adding β-amyrin. Subsequently, they were incubated for 2 h at 37°C. Thereafter, the samples were centrifuged at 12,000 rpm and supernatant were removed. Then, 1 ml PBS and 1 μl of DiBAC_4_[(3) 5 10 μg/ml] were added. The fluorescence intensity was measured using an FACSVerse flow cytometer (BD Biosciences, USA).

### Measurement of Caspase-Like Protein

CaspACE FITC-VAD-FMK In Situ Marker (Promega, USA) was used to detect caspase-like protein. *E. coli* cells (1 × 10^6^ cells/ml) were treated with β-amyrin (5 μM) or norfloxacin (5 μM), and cell some samples were pretreated with thiourea (150 mM) before adding β-amyrin to the cells. Thereafter, they were incubated for 2 h at 37°C and centrifuged at 12,000 ×*g* for 5 min, following which the supernatant was removed. Next, the cells were washed twice with PBS before adding 2.5 μM CaspACE FITC-VAD diluted with dimethyl sulfoxide. The mixture was allowed to react for 10 min and after reaction, analyzed using the FACSVerse flow cytometer.

### DNA Fragmentation Assessment

Cleavage of DNA into single stranded-DNA was assessed using terminal deoxynucleotidyl transferase dUTP nick-end labeling (TUNEL) assay (Roche Applied Science, Switzerland). Briefly, *E. coli* cells (1 × 10^6^ cells/ml) were treated with β-amyrin (5 μM) or norfloxacin (5 μM), and some cell samples were pretreated with thiourea (150 mM) before adding β-amyrin. Subsequently, they were incubated for 2 h at 37°C and centrifuged at 12,000 ×*g* for 5 min, following which the supernatant was removed. Next, 2% paraformaldehyde was added to the samples, which were then incubated for 1 h, and 100 μl of permeabilization solution (0.1% Triton X-100 and 0.1% sodium citrate) was added. The cells were washed twice with PBS and added TUNEL mixture mixed with label and enzyme samples. Finally, the fluorescence intensity of the cells was measured with spectrofluorophotometer at wavelengths of 490 nm (excitation) and 519 nm (emission).

### Assessment of Phosphatidylserion (PS) Exposure

The presence of PS on the outer membrane of *E. coli* cells was assessed with annexin V/propidium iodide (PI) double staining (BD Biosciences Pharmingen, USA). The cells (1 × 10^6^ cells/ml) were treated as described above experiments. Next, they were incubated for 2 h at 37°C and centrifuged at 12,000 ×*g*. Subsequently, 100 μg of 1× Annexin V binding buffer and 5 μg of FITC Annexin V were added, and the resultant fluorescence was measured using a FACSVerse flow cytometer.

### Statistical Analysis

All the experiments were performed in triplicate. Data are expressed as mean ± standard deviation. For three-group comparisons, Post hoc analysis using Tukey’s test for three-group comparisons was performed with SPSS software v23 (IBM Corp., USA). Differences between each group were considered statistically significant at a *p* value of <0.1, <0.05, and <0.01. It was used to prove the reliability of experiments’ results.

## Results

### β-Amyrin Induced ROS Production

**Fig. 1:**
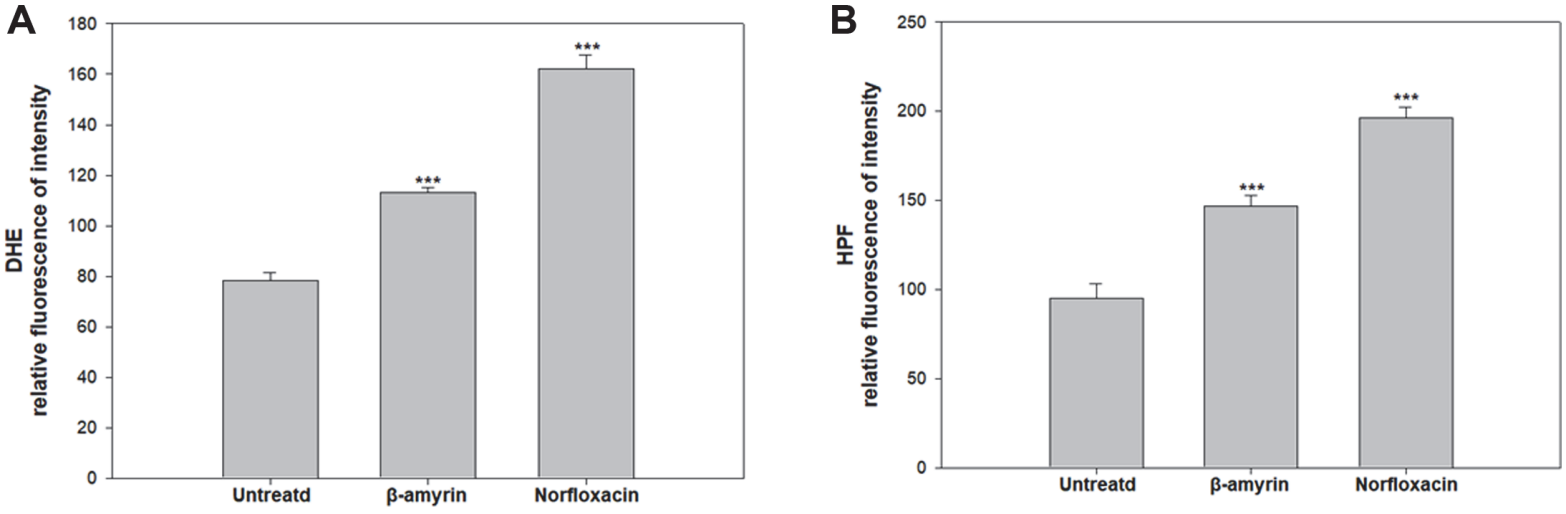
Measurement of ROS generation in *Escherichia coli*. (**A**) DHE (**B**) HPF assay were used in these experiments. DHE and HPF dye were used to detect O_2_ - or OH- detection. *E. coli* were treated with β-Amyrin (5 μM) or norfloxacin (5 μM). The experiments were conducted in triplicate. The average standard deviation was represented. (*p < 0.1; **p < 0.05; ***p < 0.01)

β-Amyrin is one of the triterpenes that induces ROS production in microbes [5]. Treatment of HPF and DHE dyes detect accumulation of OH^−^ and O_2_^−^ in the cells, respectively showing fluorescence. The cells treated with β-amyrin or norfloxacin showed a high fluorescence intensity compared with untreated cells in both experiments (Fig. 1). The cells treated with β-amyrin showed a relative intensity of 113.12 and 147.12, respectively, whereas the untreated cells showed a relative intensity of 78.21 and 95.12, respectively, indicating that β-amyrin treatment induced the production of ROS, which are toxic to cells. Finally, the cells treated with norfloxacin which is well known antibacterial agent showed 162.31 and 196.12 [16].

### Destruction of the Bacterial Defense System

**Fig. 2:**
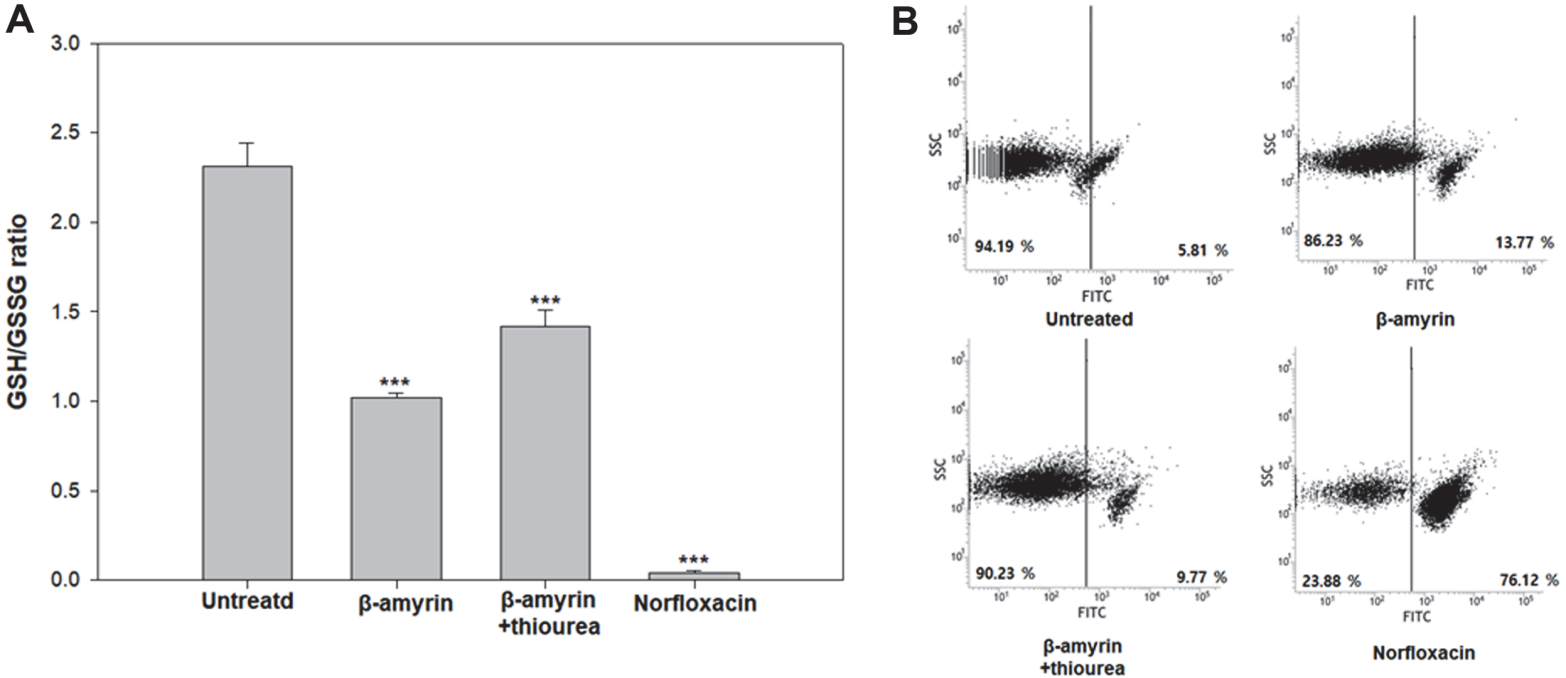
Oxidation measurement through GSH/GSSG ratio and membrane depolarization. (**A**) Glutathione assay (**B**) DiBAC_4_(3) assay were used in these experiments to check impact of ROS on bacterial defense system. *E. coli* were treated with βAmyrin (5 μM) or norfloxacin (5 μM) and some of them were pretreated with thiourea (5 μM). The experiments were conducted in triplicate. The average standard deviation was represented. (*p < 0.1; **p < 0.05; ***p < 0.01)

GSH takes a defensive role in protecting bacteria from ROS and osmotic stresses. However, excessive oxidative damage can induce GSH dysfunction. The ratio of GSH:GSSG is a marker of the degree of GSH oxidation. The cells treated with β-amyrin or norfloxacin showed ratios of 1.02 and 0.04, respectively, indicating degrees of oxidation of cell. The untreated and thiourea-pretreated cells showed significantly higher ratios of 2.31 and 1.42, respectively (Fig. 2A). These results indicated that ROS production induced by β-amyrin resulted in excessive oxidative stress in bacteria.

### Depolarization of the Bacterial Membrane

ROS production in eukaryotic cells has been explained to induce changes in the cell membrane potential [17]. Additionally, recent studies have reported a similar loss in membrane potential in bacteria. To enhance our understanding of membrane depolarizations, we treated the *E. coli* cells with DiBAC_4_(3). The cells treated with β-amyrin or norfloxacin showed high levels of relative fluorescent intensities respectively 13.77% and 76.12%, whereas the untreated and thiourea-pretreated cells showed low levels of fluorescence intensity specifically 5.81% and 9.77%(Fig. 2B). Our findings indicated that β-amyrin induced membrane depolarization, which could lead to cell death.

### Activation of Caspase-Like Protein

**Fig. 3:**
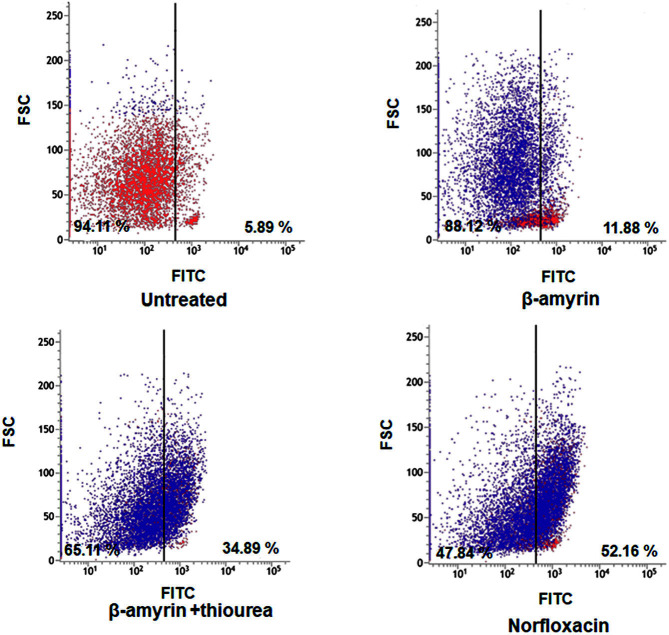
Caspase-like protein detection in *E. coli*. caspACE FITC-VAD-FMK were used in this experiment to observe caspase-like protein. *E. coli* were treated with β-Amyrin (5 μM) or norfloxacin (5 μM) and some of them were pretreated with thiourea (5 μM). The experiments were conducted in triplicate. The average standard deviation was represented. (*p < 0.1; **p < 0.05; ***p < 0.01)

The correlation between caspase-like protein and ROS has been investigated [18]. Apoptosis is mediated by a family of cysteine proteases called caspases which can be detected by fluorescence probe. When the substrate is released, generation of light were occurred and it were detected in a specific emission [19]. ROS stimulate cell death pathways, leading to caspase activation. Therefore, we assessed the presence of caspase-like proteins in bacteria to understand under which conditions caspase activation occurred. The cells treated with β-amyrin or norfloxacin showed caspase activation in 34.89% and 52.16% of the population, respectively, whereas the thiourea-pretreated and untreated cells showed a lower percentage 11.88 % and 5.89 %, respectively) of caspase activation in the cell populations (Fig. 3).

### DNA Fragmentation

**Fig. 4:**
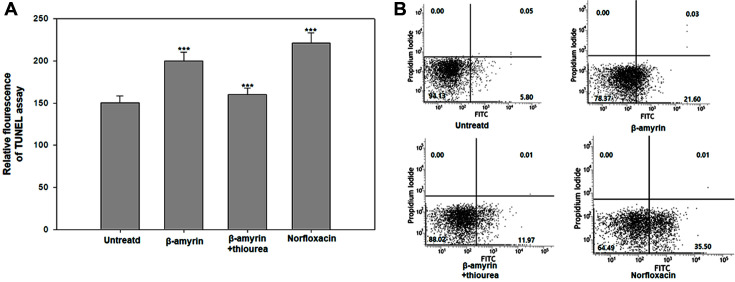
DNA fragmentation and Annexin/PI double staining detection. (**A**) TUNEL assay (**B**) Annexin/PI double staining were used to detect DNA fragmentation and apoptosis-like death in *E. coli*. Cells were treated with with β-Amyrin (5 μM) or norfloxacin (5 μM) and some of them were pretreated with thiourea (5 μM). The experiments were conducted in triplicate. The average standard deviation was represented. (*p < 0.1; **p < 0.05; ***p < 0.01)

Because activated caspase stimulates endonuclease, which is related to the DNA fragmentation, the fragmentation of DNA into single strands is a hallmark of apoptosis-like death [20, 21]. We used the TUNEL assay to check DNA fragmentation [22]. This assay was designed to label blunt ends of double-stranded DNA breaks independent of a template [23]. The cells treated with β-amyrin or norfloxacin showed high relative fluorescence intensity (199.98 and 220.95, respectively), whereas the untreated and thiourea-pretreated cells showed a low relative fluorescence intensity (150.23 and 160.09, respectively) (Fig. 4). These results indicate that β-amyrin induced DNA cleavage.

### PS Exposure

The bacterial cell membrane prevents the leakage of proteins or ions from cells, and PS is usually located in the inner side of the membrane [24]. However, when cells undergo apoptosis-like death, PS becomes exposed to the outer membrane. Thus, the binding of annexin V to PS on the outer membrane indicates apoptosis-like death. PI, which is a membrane-impermeable nucleic acid stain, sticks to the nucleus of necrotic cells [25]. The cells treated with β-amyrin and norfloxacin were stained positive for annexin V and negative for PI (Fig. 4). The results means that β-amyrin caused apoptosis-like death in *E. coli*.

## Discussion

Pentacyclic triterpenes are widespread throughout the plant kingdom. These active phytochemicals have various properties, including anti-inflammatory and antitumor effects [26, 27]. In particular, β-amyrin, which has a pentacyclic structure, exhibited antifungal effects by inducing ROS production [5]. However, the antibacterial effect of pentacyclic triterpenes remains unclear. Thus, we focused on the mechanisms underlying the antibacterial effect of β-amyrin.

The correlation between ROS and cell function has been established [28]. Oxidative damage exerted by ROS on bacteria induces protein inactivity and leads to cell death [29]. ROS are highly reactive molecules and include diverse species, such as O_2_^−^, OH^−^, and H_2_O_2_. Specifically, O_2_^−^ is critical for the production of OH^−^ and H_2_O_2_. The stepwise reduction of O_2_ to H_2_O involves several ROS [30] and results in the generation of O_2_^−^, leading to OH^−^ production, which is extremely destructive to bacterial components. The most reactive molecular in ROS, OH^−^ accumulation in bacteria can lead to the several dysfunctions and death [31].

Our investigations revealed that β-amyrin induced ROS production, which resulted in oxidative stress by destroying bacterial defense systems. Therefore, ROS generation is the main effector of the antibacterial effects of β-amyrin. To investigate the specific role of ROS, we treated *E. coli* cells with thiourea, an ROS scavenger of OH^−^, to prevent apoptosis-like death following β-amyrin treatment. Oxidative stress leads to the inactivation of proteins, such as GSH. GSH is important for protecting cells from lethal situations such as oxidation and osmotic stresses [32] and is a major antioxidant that exerts protective effects against ROS-mediated DNA damage and apoptosis [14]. Decrease of the ratio (GSH:GSSG) means GSH dysfunction, resulting in the dysfunctions of the bacterial defense system and oxidative stress caused by ROS cannot be dismuted.

When GSH levels become depleted, the bacterial cells are no longer protected against oxidation and osmotic stresses, leading to membrane depolarization and changes in membrane potential. Uncontrolled ROS in bacteria causes secondary damages like depolarization [33]. Usually, the cytoplasmic membrane protects bacteria from antimicrobial compounds. However, ROS can alter the membrane potential, thereby disrupting its essential barrier function [34], and this can be verified by detecting changes in membrane potential. When bacteria experience excessive oxidative stress leading to membrane depolarization, it results in cell death [35].

Caspase is linked with regulatory networks and controlled inflammation and cell death in eukaryote. Caspases are crucial mediators of apoptosis in eukaryotes especially in yeasts. However, caspase-like proteins were demonstrated in previous study and elucidated to induce apoptosis-like death in bacteria by activating apoptotic signaling pathways [11, 36]. Moreover, Caspase-like proteins in bacteria are recently detected when cells undergo programmed cell death [36]. Therefore, our study findings revealed that β-amyrin activated caspase-like proteins in bacteria, which could lead to apoptosis-like death in bacteria.

Bacterial cells undergo apoptosis-like death show common characters such as DNA fragmentation and PS exposure. When apoptosis-like death occurred, chromosomal DNA is cleaved into oligonucleosomal-sized fragments by caspase-like proteins leading to the cell death [20, 37]. Thus, fragmented DNA is the main feature of apoptosis-like death in bacteria. PS exposure is a feature of apoptotic cells as well as a signal of nonapoptotic cell death [38]. To obtain further insight, we used double staining with annexin V and PI to distinguish between apoptosis-like death and necrosis. β-amyrin triggered the cells positive for annexin V and negative for PI (*i.e.*, they were apoptotic).

In summary, this paper focused on explaining the antibacterial mode of action of β-amyrin. We treated *E. coli* cells with β-amyrin, resulting in ROS accumulation. The production of O_2_^−^ is not very toxic to bacteria itself, but OH^−^, which forms during the reduction of O2, is lethal to bacteria. OH^−^ has high molecular reactivity, leading to bacterial protein oxidation and GSH dysfunction. GSH depletion induces the destruction of the bacterial defense system via oxidation and alterations in membrane potential. The resultant depolarization of the cell membrane destroys its essential barrier function and triggers cell death. Cell death, particularly apoptosis-like death, has common characteristics such as caspase-like protein activation and fragmentation of DNA. Caspase-like proteins initiate apoptotic signaling and are a marker of apoptosis. Moreover, caspase-like protein is considered mediators that stimulate hydrolytic enzyme-mediated degradation and induce DNA fragmentation. Therefore, the antibacterial mode of action of β-amyrin involves the induction of apoptosis-like death via ROS accumulation.
